# Integrated ESR and PLT‐H Measurement Using the BC‐6800 Plus Hematology Analyzer: A Comprehensive Analytical Evaluation

**DOI:** 10.1002/jcla.70185

**Published:** 2026-02-20

**Authors:** Meilin Chen, Jinghai Yan, Manman Ye, Shihong Zhang, Xiaohe Zheng

**Affiliations:** ^1^ Department of Laboratory Medicine The First Affiliated Hospital, Sun Yat‐Sen University Guangzhou China

**Keywords:** analytical performance, erythrocyte sedimentation rate (ESR), hematology analyzer, Mindray BC‐6800 plus, platelet hybrid count (PLT‐H)

## Abstract

**Background:**

Complete blood count (CBC) and erythrocyte sedimentation rate (ESR) are essential tests in clinical practice. The Mindray BC‐6800 Plus integrates CBC and ESR analysis through its Easy‐W ESR module and a hybrid platelet channel (PLT‐H) designed to improve platelet quantification, especially under interference.

**Methods:**

A prospective analytical study was conducted following CLSI guidelines (EP05‐A3, EP06‐A, EP09c). A total of 460 venous samples were analyzed across multiple modes (Auto, Open‐Vial, Predilute). PLT‐H was compared with PLT‐O and reference values; ESR with the BC‐760CS, which shares the same ESR module validated against Westergren. Interference testing included samples with low MCV (< 75 fL) and high MPV (> 10 fL).

**Results:**

PLT‐H background was consistently < 5 × 10^9^/L. Precision met predefined limits (CV ≤ 4% whole blood, ≤ 8% predilute; ESR SD ≤ 2 mm/h at ≤ 20 mm/h, CV ≤ 10% at > 20 mm/h). Carryover was ≤ 0.2%. PLT‐H showed excellent linearity to 5000 × 10^9^/L (*r* = 0.999) and tighter bias distribution than PLT‐O. Agreement was strong for ESR between BC‐6800 Plus and BC‐760CS (*r* = 0.989) and for PLT‐H versus PLT‐O (*r* = 0.992). PLT‐H maintained robustness in interference‐prone samples (*r* = 0.992).

**Conclusion:**

The BC‐6800 Plus demonstrated excellent analytical performance for ESR and PLT‐H, delivering accurate, reproducible, and interference‐resistant results. It supports efficient, high‐quality testing in modern laboratories.

## Graphical abstract

This study evaluated the analytical performance of the Mindray BC‐6800 Plus hematology analyzer, focusing on integrated erythrocyte sedimentation rate (ESR) and hybrid platelet counting (PLT‐H). Results demonstrated excellent background suppression, precision, linearity, and robustness across different modes and analyzers, confirming its reliability for consolidated inflammatory and hematologic testing.

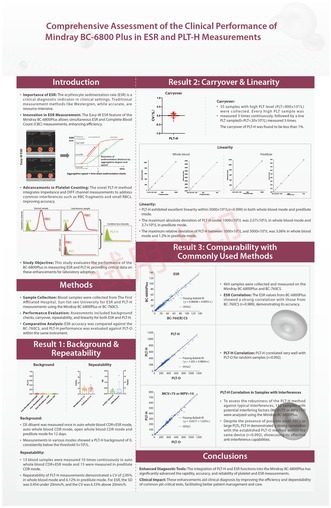

## 1 Introduction

The complete blood count (CBC) and erythrocyte sedimentation rate (ESR) are among the most frequently requested laboratory tests in clinical practice [1, 2], playing essential roles in diagnosing and monitoring infections [3, 4], inflammatory diseases [5, 6], hematologic disorders [7], and malignancies [8]. Traditionally, these two tests are performed separately—CBC via hematology analyzers and ESR via manual or semiautomated methods such as the Westergren technique [2]. This separation introduces additional preanalytical steps, prolongs turnaround time, and increases the risk of sample mismanagement, particularly in high‐throughput laboratories.

The Mindray BC‐6800 Plus hematology analyzer introduces a novel integration of CBC and ESR testing through its Easy‐W ESR module. This module analyzes erythrocyte aggregation behavior in real time, estimating sedimentation dynamics using curve‐derived parameters such as aggregation degree and aggregation speed (as illustrated in Figure S1). This approach enables ESR measurement without requiring additional blood volume or reagents. Specifically, the minimum sample requirement is 1.0 mL for automated modes and 0.5 mL for open‐vial/prediluted modes, which is comparable to or lower than most dedicated ESR analyzers. Importantly, the Westergren method remains the reference standard recommended by the ICSH and CLSI, and any automated ESR evaluation should be benchmarked against it [9].

In parallel, the analyzer incorporates an innovative platelet measurement method: PLT‐H. This hybrid parameter combines data from the impedance channel (ideal for detecting small platelets) and the DIFF optical channel (which accurately identifies large platelets) to correct for common interferences such as RBC fragments or microcytic cells. As illustrated in Figure S2, this dual‐channel integration allows the system to distinguish platelets more effectively in both normal and interference‐prone samples, improving overall platelet counting accuracy without requiring dedicated fluorescent reagents.

Although the BC‐6800 Plus is capable of performing the entire CBC panel, the present study specifically focused on ESR and PLT‐H. This focus was chosen because (1) ESR remains a widely used but under‐validated parameter in automated hematology systems, and (2) PLT‐H represents a novel algorithm intended to address long‐standing challenges in platelet enumeration. Other CBC parameters of the analyzer have been validated extensively in previous studies and manufacturer reports, whereas ESR and PLT‐H together represent key areas where additional evidence is required for real‐world laboratory practice.

While prior studies have evaluated platelet measurement accuracy in isolated contexts [10], the combined performance of ESR and PLT‐H in real‐world clinical scenarios remains underreported. Automated hematology analyzers adopt various platelet counting techniques, each with inherent strengths and limitations [11]. The most commonly used approach, impedance‐based platelet counting (PLT‐I), classifies particles by size but lacks specificity for distinguishing platelets from similarly sized elements such as small red blood cells (RBCs), RBC fragments, or white blood cell (WBC) debris [12, 13]. These interferences can lead to falsely elevated or reduced platelet counts—for example, RBC fragments and microcytes may mimic platelets, while giant platelets or platelet aggregates may be undercounted [14].

To overcome these challenges, many platforms have implemented optical counting methods (PLT‐O), such as those found in the Abbott CELL‐DYN and Siemens ADVIA series, which improve the identification of large platelets but remain susceptible to interference from small RBCs or cellular debris [14]. More advanced fluorescent optical technologies—used in systems like the Sysmex XE/XN series and Mindray BC‐6000/700/6800 series—leverage nucleic acid dyes to enhance platelet recognition, particularly in thrombocytopenic samples [15, 16]. However, these methods require dedicated physical channels (e.g., RET) and additional reagents, increasing operational costs.

As a cost‐efficient alternative, Mindray's BC‐700 and BC‐6800 series have introduced the hybrid platelet count parameter (PLT‐H) [17]. This method combines data from the impedance channel (for small platelets) and the DIFF optical channel (for large platelets), providing improved accuracy without the need for extra reagents or hardware.

Moreover, the BC‐6800 Plus allows flexible operation across various sampling modes—including auto‐loaded, open‐vial, and prediluted configurations—enhancing its adaptability to diverse laboratory environments [18]. Its integration of advanced flagging systems and multichannel measurement strategies presents an opportunity to consolidate routine hematology and inflammation monitoring onto a single platform.

In this study, we aimed to comprehensively assess the analytical and clinical performance of the Mindray BC‐6800 Plus hematology analyzer with respect to ESR and PLT‐H measurements. The evaluation covered background noise, repeatability, carryover, linearity, interinstrument and intermode consistency, interference robustness (e.g., low MCV or high MPV), and comparability with established reference methods (BC‐760CS for ESR and PLT‐O channel for platelets). This integrated analysis is intended to validate the system's reliability and demonstrate its suitability for high‐efficiency, high‐accuracy testing in modern clinical laboratories.

## 2 Materials and Methods

### 2.1 Study Design and Objective

This prospective analytical study evaluated the integrated performance of the Mindray BC‐6800 Plus hematology analyzer for erythrocyte sedimentation rate (ESR) and hybrid platelet count (PLT‐H). Study design followed CLSI recommendations: EP05‐A3/EP15‐based precision evaluation (repeatability and within‐laboratory precision) [19], EP06‐A (linearity) [20] and EP09c (method comparison) [21]. A total of 460 venous whole‐blood specimens were used to assess background, carryover, precision (repeatability), linearity, method comparison (accuracy/agreement), interference, between‐mode consistency, and sample stability.

### 2.2 Instruments

The BC‐6800 Plus (Mindray) with Easy‐W ESR and PLT‐H modules served as the primary analyzer. Platelet testing compared two channels within the same instrument (PLT‐H vs. PLT‐O) to minimize interinstrument variability and directly assess the novel PLT‐H algorithm.

For ESR, comparator testing was performed on the Mindray BC‐760CS, which incorporates the same ESR module as the BC‐720 series previously validated against the Westergren method [22]. Direct Westergren testing was not performed and is acknowledged as a study limitation.

### 2.3 Sample Collection and Preparation

Residual, de‐identified venous whole‐blood samples from outpatients and inpatients at the First Affiliated Hospital of Sun Yat‐sen University were included. Anticoagulant was EDTA‐K_2_ (1.5–2.2 mg/mL). Samples were processed within 4 h of collection [23]. Exclusion criteria: visible clotting or marked hemolysis on visual inspection or instrument alerts.

Minimum volumes: 1.0 mL (automated modes) and 0.5 mL (open‐vial/predilute).

To challenge performance, the set included samples with microcytosis (MCV < 75 fL), macroplatelets (MPV > 10 fL), and thrombocytosis (> 900 × 10^9^/L) [13, 14].

Ethics: Residual, de‐identified specimens used exclusively for analytical validation; per institutional policy, individual consent was not required and ethics approval was waived/determined not required.

### 2.4 Measurement Modes

Three operation modes were used: Auto Whole‐Blood (CDR and CDR + ESR), Open‐Vial Whole‐Blood, and Open‐Vial Predilute. Mode selection depended on specimen volume and routine laboratory workflow (e.g., predilute for pediatric/low‐volume samples [24, 25]). The same specimens were run across modes when applicable to enable between‐mode comparisons.

### 2.5 Background and Carryover Evaluation

Background levels were measured using diluent blanks across all modes. Acceptance thresholds were predefined: PLT‐H background ≤ 5 × 10^9^/L [26].

Carryover was determined by alternating high and low samples and calculating carryover as [(L1 − L3)/(H3 − L3)] × 100; the acceptance limit was ≤ 1.0% in line with CLSI guidance [21]. All carryover results were below 1%.

### 2.6 Repeatability (Precision)

Repeatability was assessed using 10 replicates at three levels for both ESR and PLT‐H.

PLT‐H: CV% computed in whole‐blood and predilute modes. Predefined goals: CV ≤ 4% (whole blood) and CV ≤ 8% (predilute), consistent with CLSI EP05‐A3 procedures and published analyzer performance [10, 18, 19].

ESR: For results ≤ 20 mm/h, repeatability was expressed as SD (goal: SD ≤ 2 mm/h). For results > 20 mm/h, repeatability was expressed as CV% (goal: CV ≤ 10%). The 20 mm/h decision level was used as the clinical partition for precision assessment [2, 7, 19, 22, 27, 28].

Within‐laboratory precision was evaluated using a multiday design consistent with CLSI EP05‐A3/EP15 principles. ESR and PLT‐H were measured over multiple consecutive days with multiple runs per day and replicate measurements per run. Components of imprecision were summarized as within‐run (repeatability) and between‐day variability, and overall within‐laboratory precision was reported as total CV% (or SD for low ESR levels) based on the variance components.

### 2.7 Linearity

To assess analytical linearity across the full reportable range (0–5000 × 10^9^/L), manufacturer‐provided platelet‐equivalent materials were used in accordance with CLSI EP06‐A recommendations. These materials were applied specifically to challenge the upper analytical limit of the system, whereas patient whole‐blood samples were emphasized for evaluation at clinically relevant concentration ranges.

Deviations were judged against predefined limits (≤ 10% at medical decision levels; ≤ 20% at extremes) [20]. For reporting consistency, concentrations < 1000 × 10^9^/L are referred to as the “low range” relative to the full 0–5000 × 10^9^/L analytical span.

### 2.8 Method Comparison

ESR: Matched specimens were tested on BC‐6800 Plus and BC‐760CS [22, 27, 29]. Agreement was evaluated using Passing–Bablok regression, Pearson correlation, and Bland–Altman analysis per CLSI EP09c [21].

Platelets: Accuracy of PLT‐H was evaluated against manufacturer‐assigned reference targets (stabilized materials) tested in duplicate. Comparative agreement between PLT‐H and PLT‐O was assessed using regression‐based analysis and distributional summaries (e.g., bias plots). A priori accuracy criterion for bias was ±20% in line with published platelet counting quality goals under interference‐prone conditions [9, 13, 14].

### 2.9 Interference Evaluation

A panel of 132 specimens with potential interferences (microcytosis, macroplatelets, thrombocytosis) was analyzed to evaluate robustness. Acceptance criterion for interference effect was predefined as relative bias ≤ 20% versus baseline/comparator, consistent with CLSI EP07 principles and GFHC guidance for platelet count quality goals [13, 14, 30].

### 2.10 Between‐Mode Consistency

Identical specimens were measured in Auto Whole‐Blood, Open‐Vial Whole‐Blood, and Predilute modes. Agreement across modes was evaluated per CLSI EP09c [21] comparison procedures. A priori analytical goal for between‐mode agreement was mean bias ≤ 10% [18, 21].

### 2.11 Sample Stability

Five venous whole‐blood specimens were used to evaluate the time‐related stability of erythrocyte sedimentation rate (ESR) and hybrid platelet count (PLT‐H) on the BC‐6800 Plus analyzer. Samples were stored at room temperature (18°C–26°C) and under refrigerated conditions (2°C–8°C) and measured at baseline (0 h) and at predefined time points up to 24 h (room temperature) or 48 h (refrigeration).

Stability was assessed by comparing results at each time point with the baseline value (0 h), and expressed as relative change (%). Prespecified acceptance criteria were ≤ 10% for PLT‐H and ≤ 15% or ≤ 3 mm/h for ESR at the 20 mm/h clinical decision level. Stability was considered acceptable when all observed changes remained within these limits.

### 2.12 Within‐Laboratory Precision

Within‐laboratory precision of erythrocyte sedimentation rate (ESR) and hybrid platelet count (PLT‐H) was evaluated using a multiday, multirun experimental design in accordance with CLSI EP05‐A3 recommendations. Whole‐blood samples covering clinically relevant concentration levels were measured in replicate runs across multiple days under routine operating conditions.

Precision components were estimated for within‐run, between‐run, and between‐day variability. For PLT‐H, imprecision was expressed as coefficient of variation (CV%). For ESR, imprecision was expressed as standard deviation (SD) for results ≤ 20 mm/h and as CV% for results > 20 mm/h, consistent with commonly accepted practice at the 20 mm/h clinical decision level [7, 19, 28].

Prespecified acceptance criteria were CV ≤ 4% for within‐run PLT‐H in whole‐blood mode and CV ≤ 8% in predilute mode; for ESR, acceptable limits were SD ≤ 2 mm/h at ≤ 20 mm/h and CV ≤ 10% at > 20 mm/h. Precision performance was considered acceptable when observed values met these criteria.

## 3 Result

### 3.1 Background and Repeatability

#### 3.1.1 Background

**FIGURE 1:**
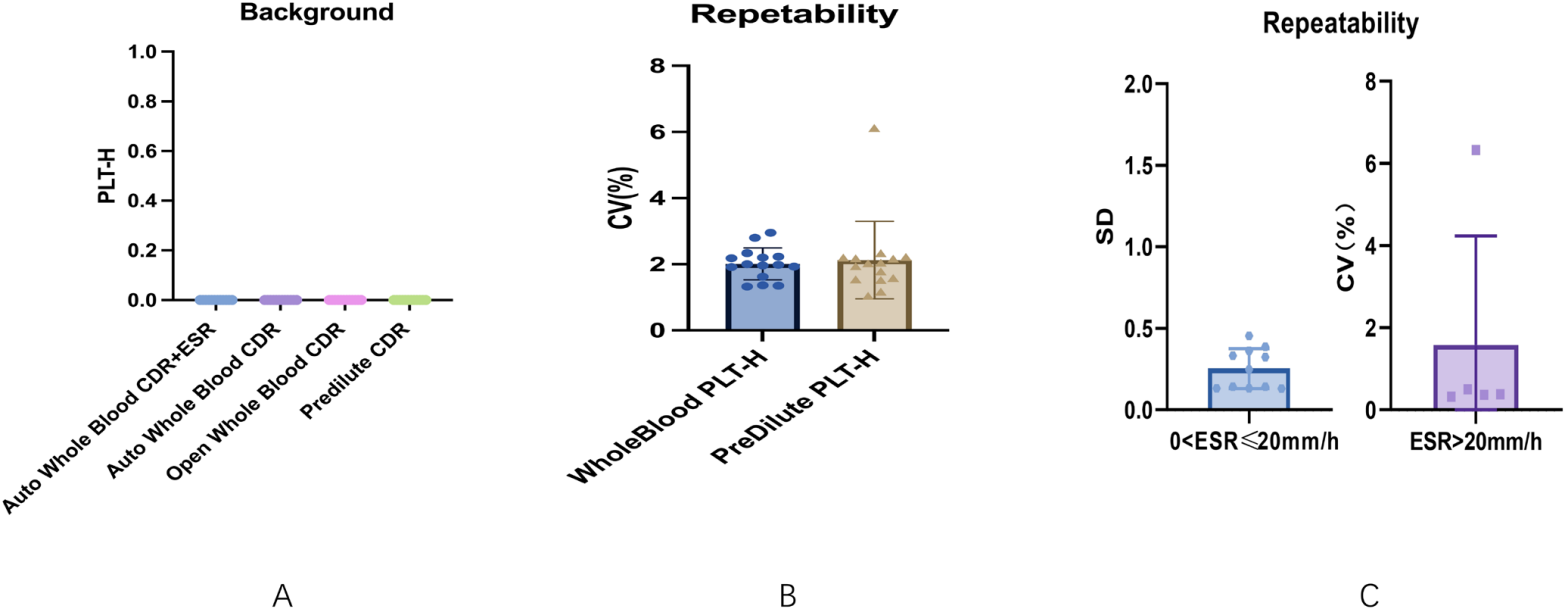
Background and repeatability of PLT‐H and ESR. (A) PLT‐H background in DS diluent blanks measured across operating modes on analyzer #3; all readings were 0 × 10^9^/L (threshold ≤ 5 × 10^9^/L). (B) PLT‐H repeatability from 10 consecutive replicates of 15 samples in whole‐blood and predilute modes (CV = 2.95% and 6.12%, respectively). (C) ESR repeatability at decision levels: SD for ≤ 20 mm/h (0.131–0.454 mm/h) and CV% for > 20 mm/h (0.32%–6.33%); all within acceptance limits.

Background tests were conducted to evaluate potential false‐positive platelet detection in DS diluent samples using the PLT‐H channel. Measurements were performed once daily over a 12‐day period on analyzer 3# across four different operating modes: Auto Whole Blood CDR + ESR, Auto Whole Blood CDR, Open Whole Blood CDR, and Predilute CDR, all modes consistently yielded a background value of 0 × 10^9^/L, far below the defined threshold of 5 × 10^9^/L (Figure 1A). To further verify these results, formal testing was conducted using four different analyzer units (1#, 3#, 5#, and 6#). As summarized in Table S1, all background values observed across all operating modes and analyzer units were 0 × 10^9^/L, well below the specification threshold of ≤ 5 × 10^9^/L. These consistent results across multiple units confirm the absence of residual signal on the PLT‐H channel, demonstrating excellent background suppression and high reliability for sensitive platelet detection.

For background testing was performed because ESR is a time‐dependent aggregation phenomenon rather than a discrete particle count. As such, there is no concept of residual count or blank background analogous to cell counting channels. Instead, ESR performance was evaluated through repeatability, stability, and method‐comparison experiments, which better reflect the analytical reliability of ESR measurement.

#### 3.1.2 Repeatability

The repeatability of PLT‐H was evaluated by measuring 15 blood samples 10 consecutive times in both Auto Whole Blood CDR + ESR mode and Predilute CDR mode. As shown in Figure 1B, the coefficient of variation (CV) for PLT‐H was 2.95% in whole‐blood mode and 6.12% in predilute mode. These results met the predefined acceptance limits of CV ≤ 4% for whole blood and CV ≤ 8% for predilute samples, based on CLSI EP05‐A3 recommendations for precision evaluation and ICSH [21, 31].

As shown in Figure 1C, the repeatability of ESR was assessed using different metrics depending on the clinical decision level. A threshold of 20 mm/h was selected to distinguish between low and high ESR values, as this level is widely cited in rheumatology and hematology practice as a clinically meaningful cutoff for differentiating normal from elevated ESR [7, 19, 22, 27, 28]. For samples with ESR ≤ 20 mm/h (*n* = 11), repeatability was expressed as standard deviation (SD). The observed SD values ranged from 0.131 to 0.454 mm/h, all well below the accepted limit of SD ≤ 2 mm/h at low ESR levels. For samples with ESR > 20 mm/h (*n* = 4), repeatability was expressed as CV%, yielding values from 0.32% to 6.33%, all within the accepted limit of CV ≤ 10%.

These results demonstrate that both PLT‐H and ESR measurements exhibit excellent repeatability across clinically relevant ranges. Additional multiunit testing (Table S2) further confirmed consistent performance across analyzers, with all SD and CV values within the specified acceptance criteria.

### 3.2 Carryover and Linearity

#### 3.2.1 Carryover

**FIGURE 2:**
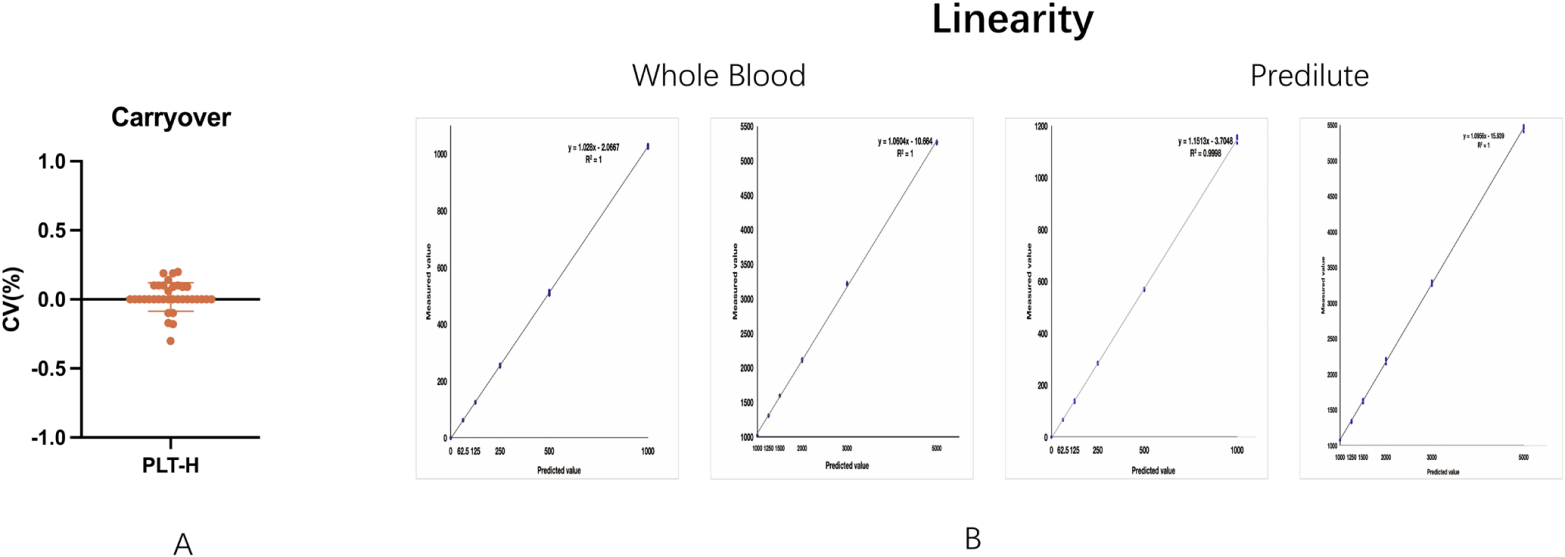
Carryover and linearity of PLT‐H. (A) Carryover across within‐mode and between‐mode sequences (range 0.00%–0.20% and −0.30% to 0.14%, respectively; limit ≤ 1.0%). (B) Linearity over 0–5000 × 10^9^/L (*r* = 0.999). Low‐range absolute deviations (< 1000 × 10^9^/L) and high‐range relative deviations (1000–5000 × 10^9^/L) were within predefined limits.

Carryover of the PLT‐H channel was evaluated to determine the extent of potential sample‐to‐sample contamination when measuring high and low platelet concentration samples sequentially. A total of 35 carryover test results were obtained, derived from 10 different testing combinations (as listed in Table S3) and all included in Figure 2A. Each test involved high platelet samples (PLT > 900 × 10^9^/L) measured three times, followed by three measurements of low platelet samples (PLT < 30 × 10^9^/L). All measurements were performed on analyzer units 4# and 6#.

Table S3 presents detailed results across both within‐mode and between‐mode scenarios. Within‐mode tests (e.g., Auto Whole Blood CDR to Auto Whole Blood CDR) yielded carryover values ranging from 0.00% to 0.20%. Between‐mode tests (e.g., Auto Whole Blood CDR + ESR to Open‐vial Predilute Whole Blood CDR) showed results from −0.30% to 0.14%. All carryover values for both analyzers were well within the predefined threshold of ≤ 1.0%, confirming that the PLT‐H channel maintains excellent performance and is free from clinically significant carryover under all evaluated conditions.

#### 3.2.2 Linearity

The linearity of the PLT‐H channel was evaluated to verify analytical performance across the reportable measurement range in whole‐blood and predilute modes. As shown in Figure 2B, PLT‐H demonstrated excellent linearity (*r* = 0.999).

To challenge the upper analytical limit of the system up to 5000 × 10^9^/L, company‐developed linearity control materials containing platelet‐equivalent particles were used. These materials provide stable and reproducible signals for analytical verification and are recommended by CLSI EP06‐A for extending linearity assessment beyond routine clinical ranges [20]. Although platelet‐equivalent particles may not fully replicate the biological behavior of native platelets, their use is appropriate for evaluating system performance at extreme analytical concentrations.

For the lower portion of the analytical span (< 1000 × 10^9^/L), absolute deviations from expected values were assessed, yielding maximum deviations of 2.07 × 10^9^/L in whole‐blood mode and 3.7 × 10^9^/L in predilute mode. Although this range exceeds the upper limit of the normal clinical reference interval (150–450 × 10^9^/L), it was defined as “low” relative to the full analytical measurement range. For higher concentrations (1000–5000 × 10^9^/L), relative deviations were evaluated, with maximum values of 3.06% in whole‐blood mode and 1.2% in predilute mode. All observed deviations remained within predefined acceptance criteria (≤ 10% at medical decision levels and ≤ 20% at extreme analytical ranges) [20].

### 3.3 Accuracy Evaluation of PLT‐H Compared to PLT‐O

To verify the analytical accuracy of PLT‐H under the Open‐Vial Whole‐Blood CDR mode, three samples with manufacturer‐assigned reference platelet values were tested in duplicate using both PLT‐H and PLT‐O channels. Relative bias (%) was calculated as:

$$\begin{array}{c} \text{Bias}\;\left(\%\right)=\left(\text{Measured value}-\text{Reference value}\right)/\text{Reference value}\\ \;\times 100. \end{array}$$

The predefined acceptance criterion was ±20%, consistent with quality recommendations for platelet counting in interference‐prone conditions. All PLT‐H results met this criterion, with relative biases ranging from −5.09% to +9.74%. PLT‐O measurements also met the acceptance criterion (−6.00% to +9.74%) but exhibited slightly greater dispersion (Table S4).

**FIGURE 3:**
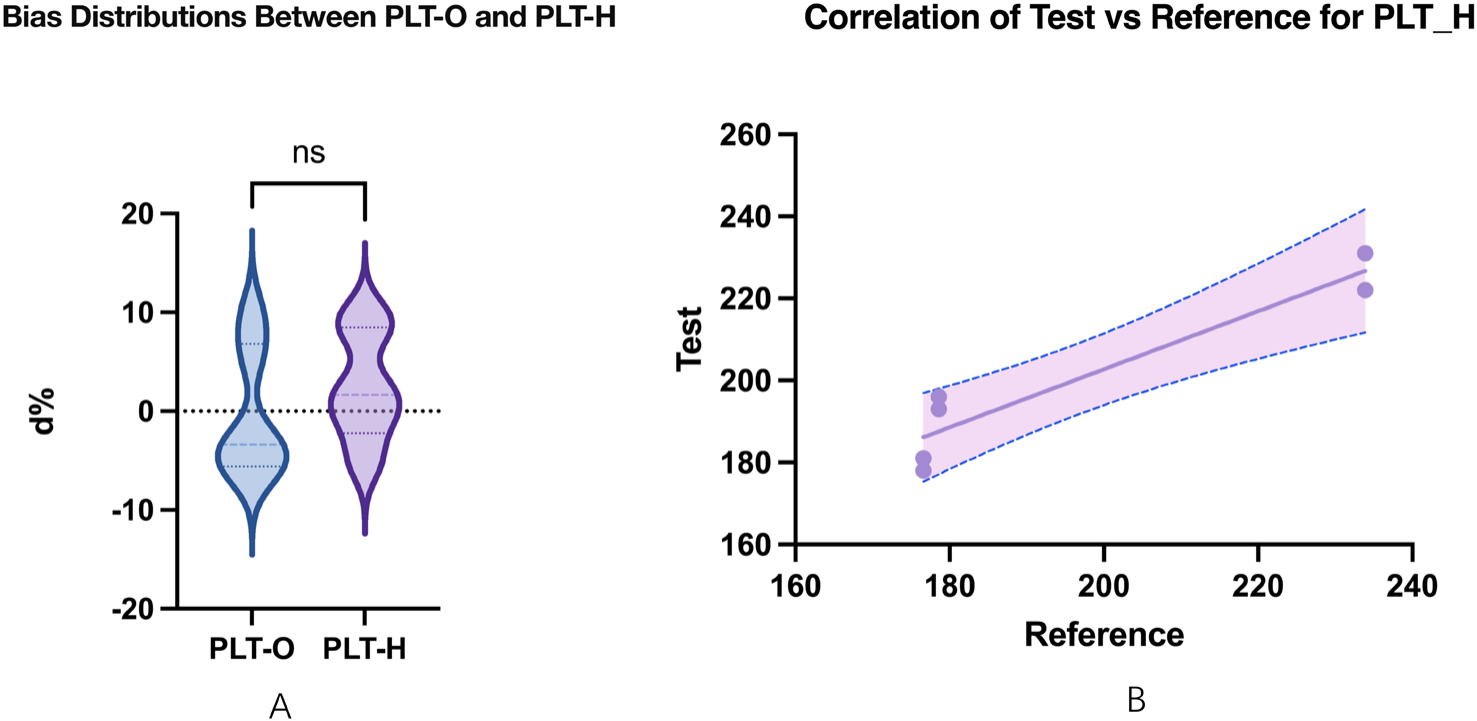
Accuracy evaluation of PLT‐H compared with PLT‐O using manufacturer‐assigned reference values. (A) Violin plots showing relative bias (%) of PLT‐H and PLT‐O measurements compared with reference platelet values under Open‐Vial Whole‐Blood CDR mode. PLT‐H biases are centered closer to zero with a narrower distribution. (B) Linear regression of measured platelet counts obtained from PLT‐H and PLT‐O channels (test methods) against manufacturer‐assigned reference values (reference). Solid lines represent the regression fits, and shaded areas indicate the 95% confidence intervals. No significant difference was observed between the two channels (*p* > 0.05).

As illustrated in Figure 3A, violin plots demonstrated that PLT‐H biases were more tightly distributed and centered closer to zero compared with PLT‐O. In Figure 3B, regression analysis of measured platelet counts (PLT‐H and PLT‐O) against manufacturer‐assigned reference values showed strong linear correlations with narrow 95% confidence intervals for both channels, with no statistically significant difference between the regression slopes (*p* > 0.05).

### 3.4 Method Comparison and Interference

#### 3.4.1 Overall ESR Correlation Between BC‐6800Plus and BC‐760CS

**FIGURE 4:**
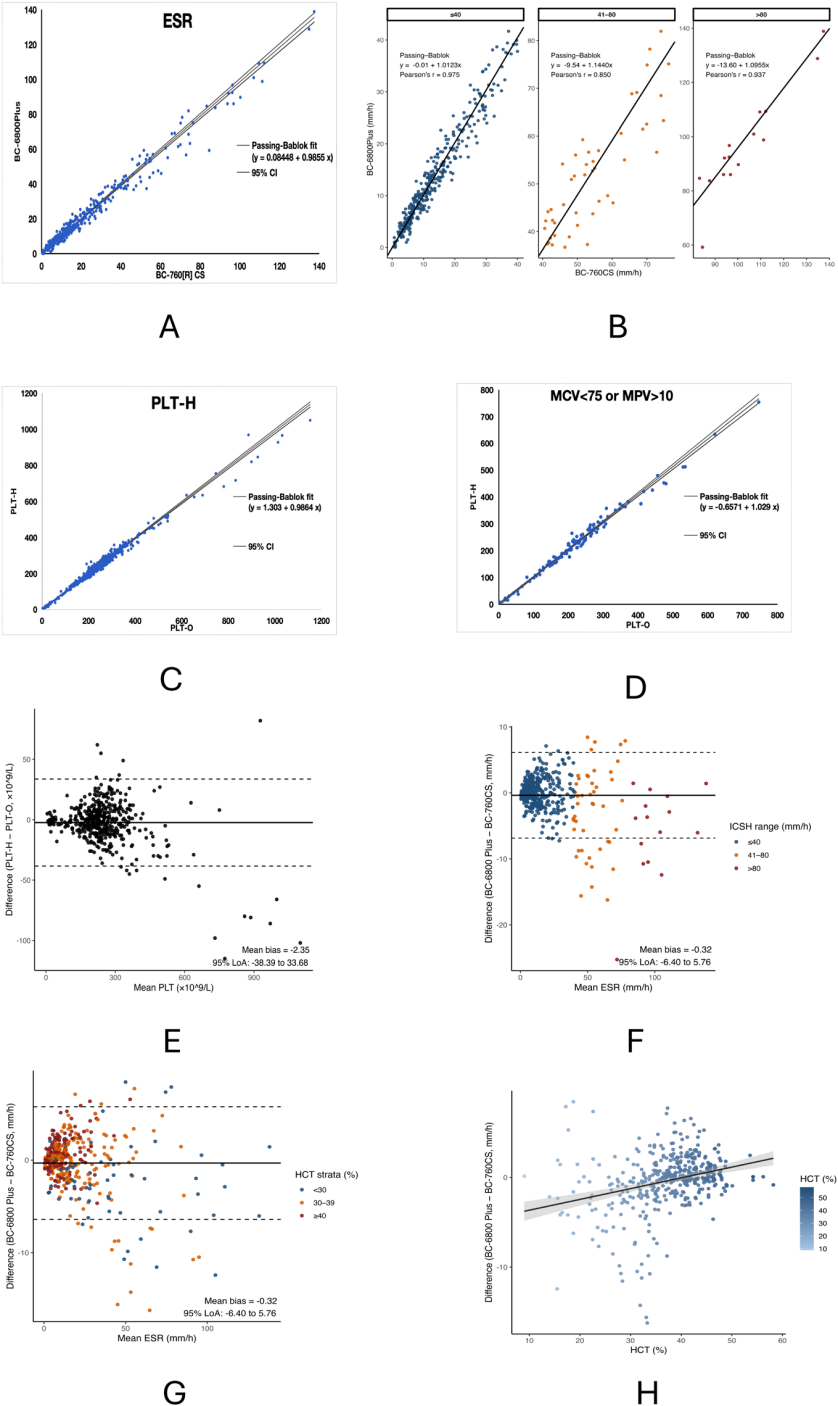
Method comparison and interference analyses. (A) Overall comparison of ESR measurements between BC‐6800 Plus and BC‐760CS using Passing–Bablok regression (*y* = 0.08448 + 0.9855*x*; *r* = 0.989). (B) Stratified Passing–Bablok regression analysis of ESR according to ICSH‐recommended ESR ranges (≤ 40, 41–80, and > 80 mm/h), with regression equations and Pearson's correlation coefficients shown in each panel. (C) PLT‐H versus PLT‐O in all samples (*y* = 1.303 + 0.9864*x*; *r* = 0.992). (D) PLT‐H versus PLT‐O in interference‐prone samples (MCV < 75 fL or MPV > 10 fL): *y* = −0.6571 + 1.029*x*; *r* = 0.992. (E) Bland–Altman analysis comparing PLT‐H and PLT‐O measurements in all samples. The solid horizontal line represents the mean bias (PLT‐H − PLT‐O), and the dashed lines indicate the 95% limits of agreement. (F) Bland–Altman analysis of ESR agreement between BC‐6800 Plus and BC‐760CS. Data points are color‐coded according to ICSH‐recommended ESR ranges (≤ 40, 41–80, and > 80 mm/h). The solid horizontal line represents the mean bias (BC‐6800 Plus—BC‐760CS), and the dashed lines indicate the 95% limits of agreement. (G) Bland–Altman plot for ESR comparison between BC‐6800 Plus and BC‐760CS, with points color‐coded by HCT strata (< 30%, 30%–39%, and ≥ 40%). Solid and dashed lines indicate mean bias and 95% limits of agreement, respectively. (H) Relationship between method difference (BC‐6800 Plus—BC‐760CS) and HCT, with linear regression fit and 95% confidence band.

A total of 460 samples were measured on both the Mindray BC‐6800 Plus and BC‐760CS analyzers to evaluate the overall agreement of ESR results. As shown in Figure 4A, Passing–Bablok regression demonstrated a strong correlation between the two systems, with a regression equation of *y* = 0.08448 + 0.9855*x* and a correlation coefficient of *r* = 0.989. The slope close to 1 and the intercept near 0 indicate excellent overall consistency between the two analyzers.

To further assess agreement across clinically relevant ESR levels, additional stratified analyses were performed according to ICSH‐recommended ESR ranges.

#### 3.4.2 Stratified ESR Agreement According to ICSH‐Recommended Ranges

According to ICSH recommendations, samples were stratified into three ESR ranges based on BC‐760CS results: ≤ 40 mm/h, 41–80 mm/h, and > 80 mm/h. Stratified Passing–Bablok regression analyses are presented in Figure 4B, and detailed stratified regression parameters are summarized in Table 1. Overall, agreement was highest in the low ESR range and remained acceptable in the intermediate and high ESR ranges.

**TABLE 1:** Stratified agreement of ESR measurements between BC‐6800 Plus and BC‐760CS according to ICSH‐recommended ranges.

ESR range (mm/h)	*n*	Regression equation (Passing–Bablok^a^)	*r*	Mean bias (mm/h)^b^	95% limits of agreement (mm/h)
≤ 40	399	*y* = 0.32 + 0.98*x*	0.975	+0.10	−4.00 to +4.19
41–80	46	*y* = 1.56 + 0.93*x*	0.850	−2.54	−15.12 to +10.04
> 80	15	*y* = −17.17 + 1.11*x*	0.937	−5.87	−19.49 to +7.76

*Note:* Limits of agreement were calculated as mean bias ±1.96 SD according to Bland–Altman analysis. ESR stratification was performed according to ICSH‐recommended clinical ranges (≤ 40, 41–80, and > 80 mm/h).

Abbreviation: ESR, erythrocyte sedimentation rate.

^a^ Regression equations were derived from Passing–Bablok analysis comparing ESR values obtained on the BC‐6800 Plus (y) and BC‐760CS (*x*).

^b^ Mean bias was calculated as ESR (BC‐6800 Plus) – ESR (BC‐760CS).

Bland–Altman analysis was additionally performed to assess agreement between ESR measurements obtained on BC‐6800 Plus and BC‐760CS. As shown in Figure 4F, the overall mean bias (BC‐6800 Plus—BC‐760CS) was small and the differences were largely distributed within the 95% limits of agreement. The Bland–Altman results for each ICSH range are provided in Table 1 (mean bias and 95% limits of agreement).

#### 3.4.3 Effect of Hematocrit (Anemia) on ESR Agreement

As shown in Figure 4G,H, a statistically significant association was observed between method difference and hematocrit (slope = 0.12 mm/h per 1% HCT, *p* < 0.001). However, the proportion of variance explained by hematocrit was small (*R* ^2^ = 0.09), indicating that hematocrit accounted for only a limited fraction of the observed method differences.

#### 3.4.4 Correlation Analysis Between PLT‐H and PLT‐O

Passing–Bablok regression was used to compare PLT‐H and PLT‐O in 460 samples. As shown in Figure 4C, PLT‐H showed strong agreement with PLT‐O (*y* = 1.303 + 0.9864*x*; *r* = 0.992).

Bland–Altman analysis further supported good agreement between PLT‐H and PLT‐O (Figure 4E), with most differences falling within the 95% limits of agreement.

#### 3.4.5 Correlation of PLT‐H in Samples With Interfering Factors

To evaluate PLT‐H performance in potentially interference‐prone samples, 132 samples with low MCV (< 75 fL) or high MPV (> 10 fL) were analyzed. As shown in Figure 4D, Passing–Bablok regression yielded *y* = −0.6571 + 1.029*x* with *r* = 0.992, indicating maintained agreement between PLT‐H and PLT‐O in this subgroup. These results indicate that even in the presence of interfering conditions such as small RBCs or large platelets, the PLT‐H method maintains a strong correlation with the conventional PLT‐O approach, confirming its robustness and reliability in challenging clinical scenarios.

### 3.5 Stability and Within‐Laboratory Precision

The time‐related stability of ESR and PLT‐H measurements on the BC‐6800 Plus analyzer was evaluated using dedicated stability samples under room temperature and refrigerated conditions for up to 48 h. As summarized in Table 2, relative changes at all evaluated time points remained within predefined acceptance criteria, indicating acceptable analytical stability.

**TABLE 2:** Time‐related stability of ESR and PLT‐H measurements on the BC‐6800 Plus analyzer.

Parameter	Storage condition	Time (h)	Maximum relative change (%)	Acceptance criterion	Acceptance met
ESR (mm/h)	Room temperature	24	7.54%	≤ 15%	Yes
PLT‐H (×10^9^/L)	Room temperature	24	7.16%	≤ 10%	Yes
ESR (mm/h)	Refrigerated (4°C)	48	2.90%	≤ 15%	Yes
PLT‐H (×10^9^/L)	Refrigerated (4°C)	48	6.82%	≤ 10%	Yes

*Note:* Time‐related stability was evaluated using whole‐blood samples stored under room‐temperature and refrigerated conditions. Relative change (%) was calculated with respect to baseline (0 h). For each analyte and storage condition, the maximum observed relative change during the evaluated period is reported. Acceptance criteria were defined according to manufacturer specifications and CLSI recommendations.

Between‐mode comparability was evaluated by comparing mode‐specific mean results and calculating absolute (d) and relative (d%) differences according to predefined specifications (Table 3). For PLT‐H, comparisons across whole‐blood CDR modes, predilute mode, and CDR + ESR mode all met the acceptance limits. For ESR, comparisons between AL Whole Blood CDR + ESR and both AL Whole Blood CD + ESR and AL Whole Blood CBC + ESR showed small differences (|*d*| ≤ 1.53 mm/h; |*d*%| ≤ 7.76%), meeting the predefined criteria (±3 mm/h or ±15%). These results indicate acceptable agreement across the evaluated measurement modes.

**TABLE 3:** Between‐mode comparison of PLT‐H and ESR results across analytical modes.

Analyte	Analyzer	Mode comparison	Mean (Mode A)	Mean (Mode B)	*d* (A − B)	*d*%	Acceptance criterion	Conclusion
PLT‐H (×10^9^/L)	3#	OA Whole Blood CDR vs. AL Whole Blood CDR	239	237	−1.67	−0.70%	±10.0%/±5%	PASS
PLT‐H (×10^9^/L)	5#	OA Whole Blood CDR vs. AL Whole Blood CDR	123	121	−2.58	−2.09%	±10.0%/±5%	PASS
PLT‐H (×10^9^/L)	3#	OA Whole Blood CDR vs. OA Predilute Mode	239	245	6.21	2.60%	±30%/±14%	PASS
PLT‐H (×10^9^/L)	5#	OA Whole Blood CDR vs. OA Predilute Mode	123	124	0.46	0.37%	±30%/±14%	PASS
PLT‐H (×10^9^/L)	3#	AL Whole Blood CDR vs. AL Whole Blood CDR + ESR	237	228	−8.78	−3.71%	±10.0%/±5%	PASS
PLT‐H (×10^9^/L)	5#	AL Whole Blood CDR vs. AL Whole Blood CDR + ESR	121	123	2.38	1.97%	±10.0%/±5%	PASS
ESR (mm/h)	3#	AL Whole Blood CDR + ESR vs. AL Whole Blood CD + ESR	17.28	17.53	0.25	1.43%	±3%/±15%	PASS
ESR (mm/h)	5#	AL Whole Blood CDR + ESR vs. AL Whole Blood CD + ESR	19.70	19.16	−0.54	−2.74%	±3%/±15%	PASS
ESR (mm/h)	3#	AL Whole Blood CDR + ESR vs. AL Whole Blood CBC + ESR	25.07	24.99	−0.09	−0.34%	±3%/±15%	PASS
ESR (mm/h)	5#	AL Whole Blood CDR + ESR vs. AL Whole Blood CBC + ESR	19.70	18.17	−1.53	−7.76%	±3%/±15%	PASS

*Note:* *d* represents the absolute difference between paired mode means (Mode A − Mode B), and *d*% represents the relative difference. Acceptance criteria (*d*/*d*%) followed the predefined specifications in the report.

Abbreviations: AL, auto analysis; CDR, complete blood count with differential; ESR, erythrocyte sedimentation rate; OA, open‐vial analysis.

Within‐laboratory precision was evaluated using a multiday, multirun design in accordance with CLSI EP05‐A3. As summarized in Table 4, both ESR and PLT‐H demonstrated acceptable within‐run and between‐day precision, supporting their suitability for routine clinical use. Operator‐related variability was not separately estimated because all measurements were performed by trained laboratory personnel following standardized operating procedures.

**TABLE 4:** Within‐laboratory precision of PLT‐H and ESR according to CLSI EP05‐A3.

Analyte	Mean level	Precision component	Observed value	Acceptance criterion
PLT‐H (whole blood)	211 × 10^9^/L	Within‐run	CV = 2.34%	CV ≤ 4.0%
PLT‐H (whole blood)		Between‐run	CV = 1.26%	CV ≤ 5.0%
PLT‐H (whole blood)		Between‐day	CV = 0.69%	CV ≤ 5.0%
PLT‐H (predilute)	53 × 10^9^/L	Within‐run	CV = 4.79%	CV ≤ 8.0%
PLT‐H (predilute)		Between‐day	CV = 2.11%	CV ≤ 10.0%
ESR (≤ 20 mm/h)	9.61 mm/h	Within‐run	SD = 0.13 mm/h	SD ≤ 2.0 mm/h
ESR (≤ 20 mm/h)		Between‐run	SD = 0.16 mm/h	SD ≤ 2.0 mm/h
ESR (≤ 20 mm/h)		Between‐day	SD = 0.00 mm/h	SD ≤ 2.0 mm/h
ESR (> 20 mm/h)	35.46 mm/h	Within‐run	CV = 1.32%	CV ≤ 10%
ESR (> 20 mm/h)		Between‐day	CV = 0.68%	CV ≤ 10%

*Note:* For ESR ≤ 20 mm/h, imprecision was expressed as SD; for ESR > 20 mm/h and PLT‐H, imprecision was expressed as CV%. Acceptance criteria were defined based on CLSI/ICSH recommendations, clinical decision levels, and commonly accepted analytical performance goals.

## 4 Discussion

The erythrocyte sedimentation rate (ESR) remains a widely used inflammatory marker. The Westergren method is still the reference approach but is labor‐intensive and sensitive to preanalytical handling [28, 32]. Automated ESR integrated in hematology analyzers seeks to mitigate these limitations by standardizing workflow.

### 4.1 ESR Performance and Interpretation

Our ESR results on the BC‐6800 Plus showed high agreement with the BC‐760CS (Passing–Bablok slope ≈1, *r* = 0.989). Prior studies reported that BC‐720/BC‐780 ESR correlates well with Westergren [22, 27]; because the BC‐760CS shares the same ESR module, our findings extend those observations to the BC‐6800 Plus under routine conditions. Importantly, we evaluated repeatability at the clinically relevant 20 mm/h decision level, a threshold frequently cited in rheumatology for distinguishing normal from elevated ESR [2, 7, 33, 34]. Precision at ≤ 20 mm/h (SD ≤ 2 mm/h) and > 20 mm/h (CV ≤ 10%) was maintained, aligning with precision‐evaluation practice [19] and supporting use in day‐to‐day monitoring. We did not perform “interference testing” for ESR because ESR is a time‐dependent aggregation/sedimentation phenomenon driven mainly by plasma proteins (e.g., fibrinogen) and RBC aggregation kinetics rather than instrumental particle‐detection artifacts; therefore, stability and method‐comparison are the most appropriate analytical checks [28, 32]. In addition, stability and between‐mode analyses demonstrated that both ESR and PLT‐H measurements on the BC‐6800 Plus were robust under routine laboratory conditions. Time‐related stability analyses demonstrated that both ESR and PLT‐H remained within predefined acceptance limits over the evaluated storage periods, indicating minimal analytical drift under routine laboratory conditions. Mode‐to‐mode differences were small and met predefined specifications, supporting consistent analytical performance across routine operating configurations. As summarized in Table 1, stratified analysis according to ICSH‐recommended ESR ranges further demonstrated that agreement between BC‐6800 Plus and BC‐760CS was highest at low ESR levels and remained acceptable at intermediate and high ranges, consistent with previously reported behavior of automated ESR methods. Notably, visual inspection of the Bland–Altman plot (Figure 4F) indicated that at higher ESR values, the BC‐760CS tended to yield slightly higher results than the BC‐6800 Plus. This systematic difference is consistent with prior reports showing increased dispersion and method‐dependent bias at elevated ESR levels, where sedimentation kinetics become increasingly nonlinear and more sensitive to plasma protein composition, red blood cell aggregation strength, and curve‐fitting assumptions used by automated ESR algorithms [28, 32]. Because both analyzers derive ESR values indirectly from optical and kinetic measurements rather than direct Westergren sedimentation, small systematic differences at high ESR levels are expected and have been documented in comparisons among automated ESR systems.

Although a statistically significant association between hematocrit and ESR method difference was observed, this analysis incorporated samples spanning a broad range of hematocrit values encountered in routine practice. The effect size was small, with hematocrit explaining less than 10% of the total variability. In practical terms, a 10% change in hematocrit corresponded to an approximate 1.2 mm/h change in method difference, which is minor relative to the overall limits of agreement. These findings suggest that while hematocrit contributes to ESR variability, its impact on method comparability between BC‐6800 Plus and BC‐760CS is limited under routine clinical conditions. Accordingly, routine hematocrit‐based correction of ESR does not appear necessary for method comparison in this setting, although extreme anemia may increase variability and warrant further investigation.

### 4.2 Platelet Counting—Why PLT‐H Matters

Platelet enumeration is prone to well‐known analytical pitfalls. Impedance‐only methods may misclassify small RBCs, fragments, or debris as platelets, whereas optical methods can underestimate counts in the presence of macroplatelets or aggregates [13, 14]. The PLT‐H algorithm on BC‐6800 Plus integrates impedance (sensitive to small platelets) with DIFF optical information (better for large platelets), aiming to reduce these error modes without additional fluorescent reagents.

In our study, PLT‐H exhibited tighter bias distributions and comparable or slightly improved agreement with reference values compared with PLT‐O, while both satisfied a conservative ±20% accuracy goal consistent with published quality recommendations for platelet counting, especially under interference‐prone conditions [13, 14]. These observations are concordant with recent evaluations showing improved accuracy/robustness of hybrid platelet approaches on the BC‐6800 Plus [10] and broadly consistent analytic performance across Mindray's current platforms [18].

At high platelet concentrations (> 600 × 10^9^/L), Bland–Altman analysis revealed a tendency for PLT‐O to yield slightly higher values than PLT‐H. This pattern is consistent with known characteristics of optical platelet‐counting methods, in which coincidence effects, signal overlap, or nonspecific particle classification may become more prominent as particle density increases. By incorporating impedance‐based constraints, the PLT‐H algorithm may limit such overestimation, resulting in a more conservative and analytically stable count in the extreme thrombocytosis range. Similar method‐dependent behavior at extreme platelet concentrations has been described in previous comparative studies of optical versus impedance‐based or hybrid platelet‐counting approaches [10, 18].

Importantly, the observed differences between PLT‐H and PLT‐O remained within predefined analytical acceptance limits and did not affect agreement across clinically relevant decision thresholds. Together with minimal background signal and negligible carryover, these results support the reliability of PLT‐H for routine platelet enumeration across a broad analytical range, including interference‐prone and high‐count conditions.

Although the BC‐6800 Plus is capable of a full CBC panel, our evaluation focused specifically on platelet parameters (PLT‐H) because this channel represents a newly developed algorithm that has not been systematically validated in real‐world settings. Other CBC parameters (e.g., WBC, RBC, Hb) have already been extensively assessed and benchmarked in previous publications, whereas PLT‐H addresses a clinically relevant gap in accurate platelet enumeration under interference‐prone conditions. Thus, emphasizing PLT‐H in this study allowed a more targeted evaluation of its analytical contribution beyond conventional CBC.

### 4.3 Interference and Robustness

In challenging samples (MCV < 75 fL or MPV > 10 fL), PLT‐H maintained correlation with PLT‐O (*r* = 0.992) and met a prespecified interference limit (≤ 20% relative bias), in line with CLSI EP07 principles. This directly addresses common analytical challenges in which microcytosis may cause false elevation with impedance methods and macroplatelets may lead to undercounting with optical approaches, scenarios where hybrid strategies can offer improved resilience [13, 14]. The background close to zero and carryover ≤ 0.2% further reduce the risk of spurious low‐count errors, which is operationally important in critical care and oncology settings.

It should be noted that these interference analyses focused on cell size–related effects and did not specifically target platelet clumping or EDTA‐induced pseudothrombocytopenia as independent conditions. Therefore, the present findings support robustness under common morphological interferences encountered in routine practice, while extreme aggregation‐related phenomena should continue to be managed using standard laboratory safeguards, such as smear review or alternative anticoagulants.

### 4.4 Linearity and Precision in Context

PLT‐H demonstrated excellent analytical linearity (*r* = 0.999) across the evaluated measurement range, with observed deviations well within CLSI EP06‐A expectations. Repeatability targets (CV ≤ 4% in whole‐blood mode and ≤ 8% in predilute mode) were consistently met, aligning with manufacturer specifications and previously reported analytical performance of the BC‐6800 Plus. Together with minimal observed carryover, these findings support robust analytical performance of the PLT‐H channel across a broad platelet concentration range under routine laboratory conditions.

### 4.5 Strengths and Limitations

The strengths of this study include a multiinstrument, multimode analytical design; prespecified acceptance criteria referenced to CLSI/ICSH recommendations and published platelet‐count guidance; and targeted stress testing under recognized interference conditions.

Several limitations should be acknowledged. First, this was a single‐center analytical evaluation, and the number of samples with extreme MCV or MPV values was limited, which may underrepresent rare hematological phenotypes. Second, direct comparison of PLT‐H with the ICSH‐recommended immunological flow cytometric reference method was not performed. However, the analytical accuracy of PLT‐H relative to flow cytometric platelet counting has been reported in prior studies, including in thrombocytopenic and interference‐prone samples [35]. Accordingly, interpretation of extreme platelet concentrations should continue to consider overall clinical context and complementary laboratory findings.

Similarly, direct comparison of ESR results with the Westergren reference method was not conducted in the present study. This is acknowledged as a limitation; however, the ESR module implemented in the BC‐6800 Plus is shared with the BC‐720/BC‐760 series, which has been independently validated against the Westergren method in previous reports [22, 27]. Finally, downstream clinical outcomes were not assessed and warrant future investigation to determine whether the observed analytical performance translates into decision‐level clinical benefits.

### 4.6 Implications

Compared with previous reports focused on ESR or platelet counting alone, our integrated evaluation suggests that BC‐6800 Plus can consolidate inflammatory monitoring (ESR) and platelet enumeration on a single platform without compromising analytical quality. The combination of low background/carryover, strong precision, linearity, and interference robustness—especially of PLT‐H—supports adoption in high‐throughput laboratories seeking efficiency with clinically dependable results. Future studies incorporating the ICSH flow‐cytometric reference, particularly in severe thrombocytopenia (< 50 × 10^9^/L), are warranted. The present study focused on analytical performance rather than clinical outcome impact; future studies linking PLT‐H and ESR results to patient management decisions may further clarify their clinical utility.

## 5 Conclusion

The Mindray BC‐6800 Plus demonstrated excellent analytical performance for both PLT‐H and ESR measurements. PLT‐H showed low background, high precision, minimal carryover (< 1%), and strong linearity up to 5000 × 10^9^/L. Accuracy analysis confirmed all PLT‐H results were within ±20% of reference values, with tighter agreement and less variability than PLT‐O. Even in samples with interferences (MCV < 75 fL or MPV > 10 fL), PLT‐H maintained high correlation with PLT‐O (*r* = 0.992). ESR results also correlated strongly with BC‐760CS (*r* = 0.989). These findings support the BC‐6800 Plus as a reliable and efficient analyzer for integrated CBC and ESR testing in clinical laboratories.

## Funding

This work was supported by Wu Jieping Medical Foundation, 320.6750.2024‐06‐74.

## Supporting information


**Figure S1:** jcla70185‐sup‐0001‐FiguresS1‐S2.pdf.
**Figure S2:** jcla70185‐sup‐0001‐FiguresS1‐S2.pdf.

**Table S1:** Background results of PLT‐H across multiple analyzer units and modes.
**Table S2:** Repeatability of PLT‐H and ESR measurements across different modes and instruments.
**Table S3:** Carryover evaluation of PLT‐H across within‐mode and between‐mode testing.
**Table S4:** Accuracy of PLT‐H and PLT‐O compared to manufacturer‐assigned reference values.

## Data Availability

The data that support the findings of this study are available on request from the corresponding author. The data are not publicly available due to privacy or ethical restrictions.
