# Supplementary figures and images for: Integrated ESR and PLT‐H Measurement Using the BC‐6800 Plus Hematology Analyzer: A Comprehensive Analytical Evaluation
Source: J Clin Lab Anal. 2026 Feb 20;40(7):e70185. doi: 10.1002/jcla.70185 (PMC13052096; doi:10.1002/jcla.70185)

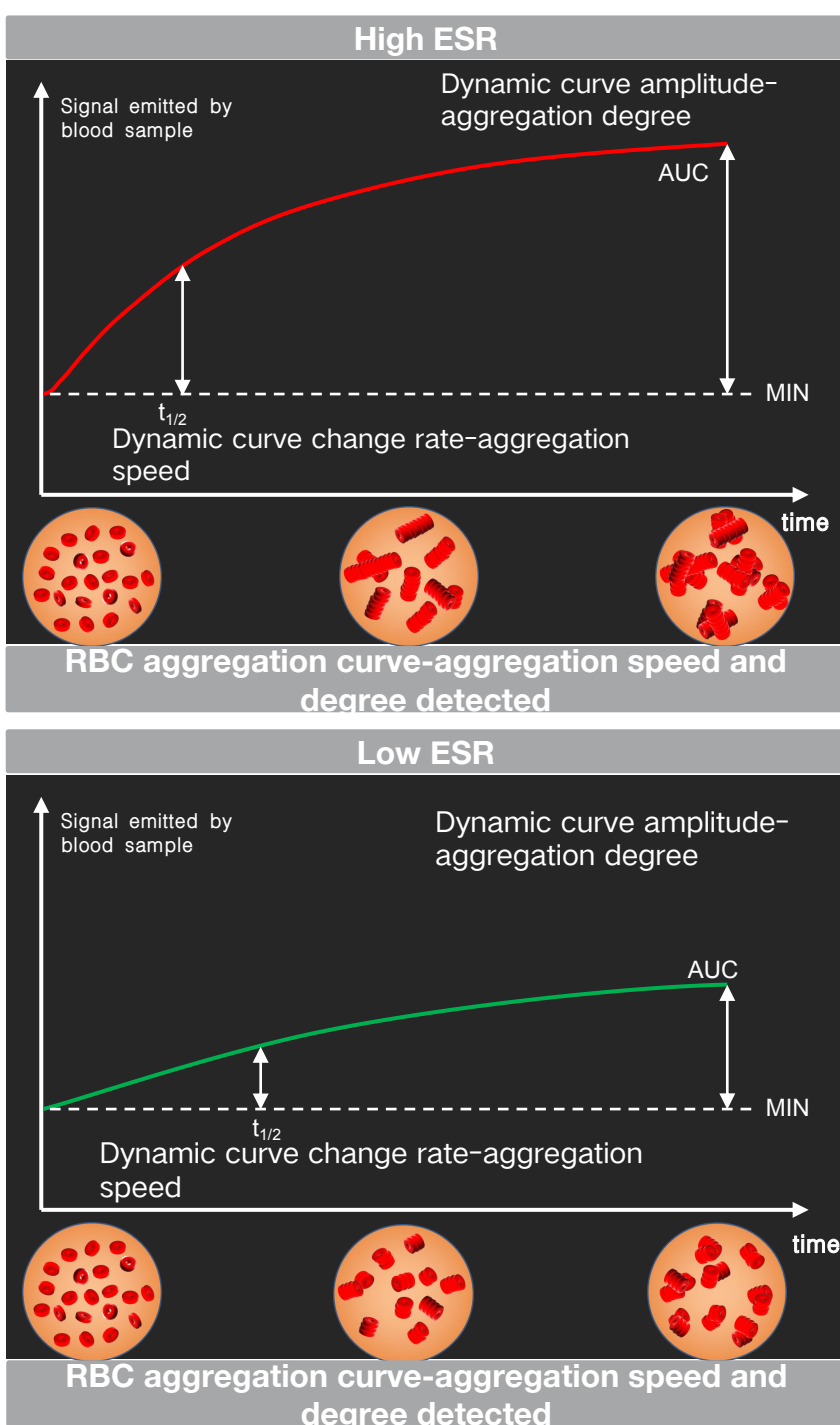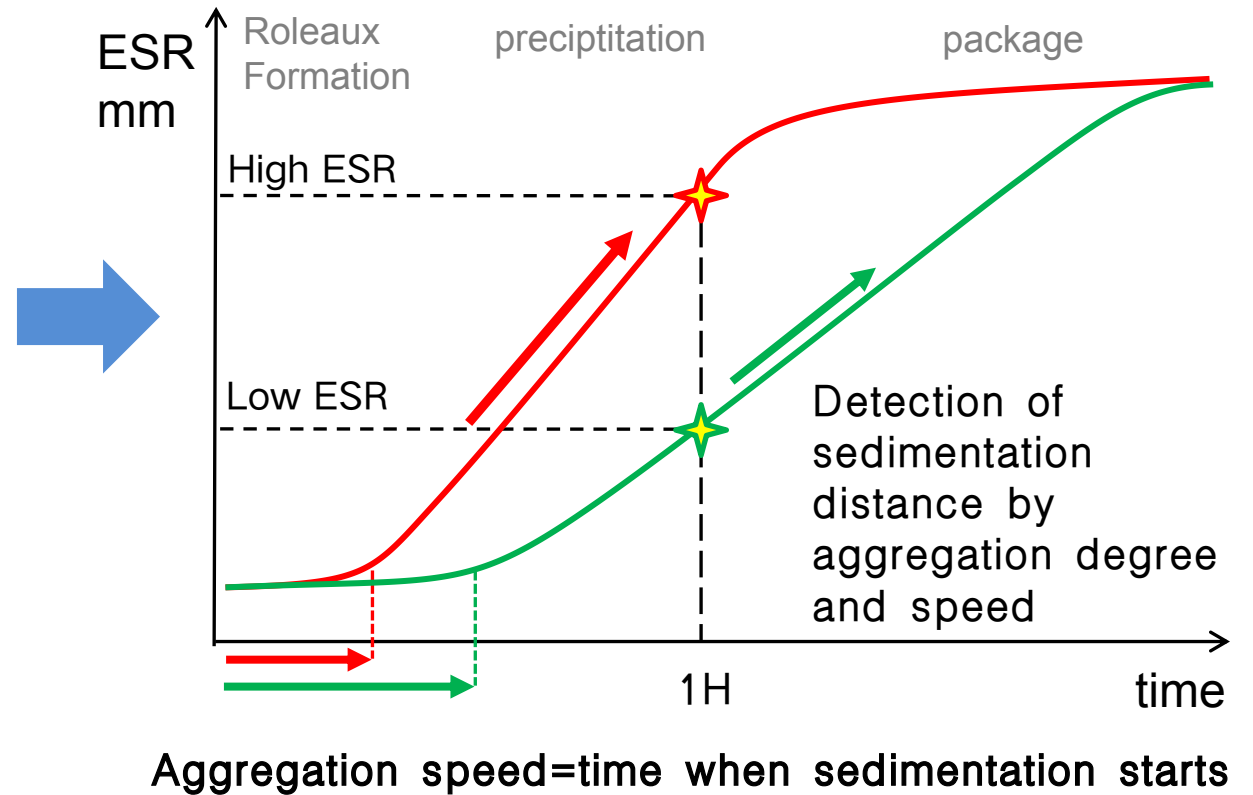

Figure 1 of the supplementary material

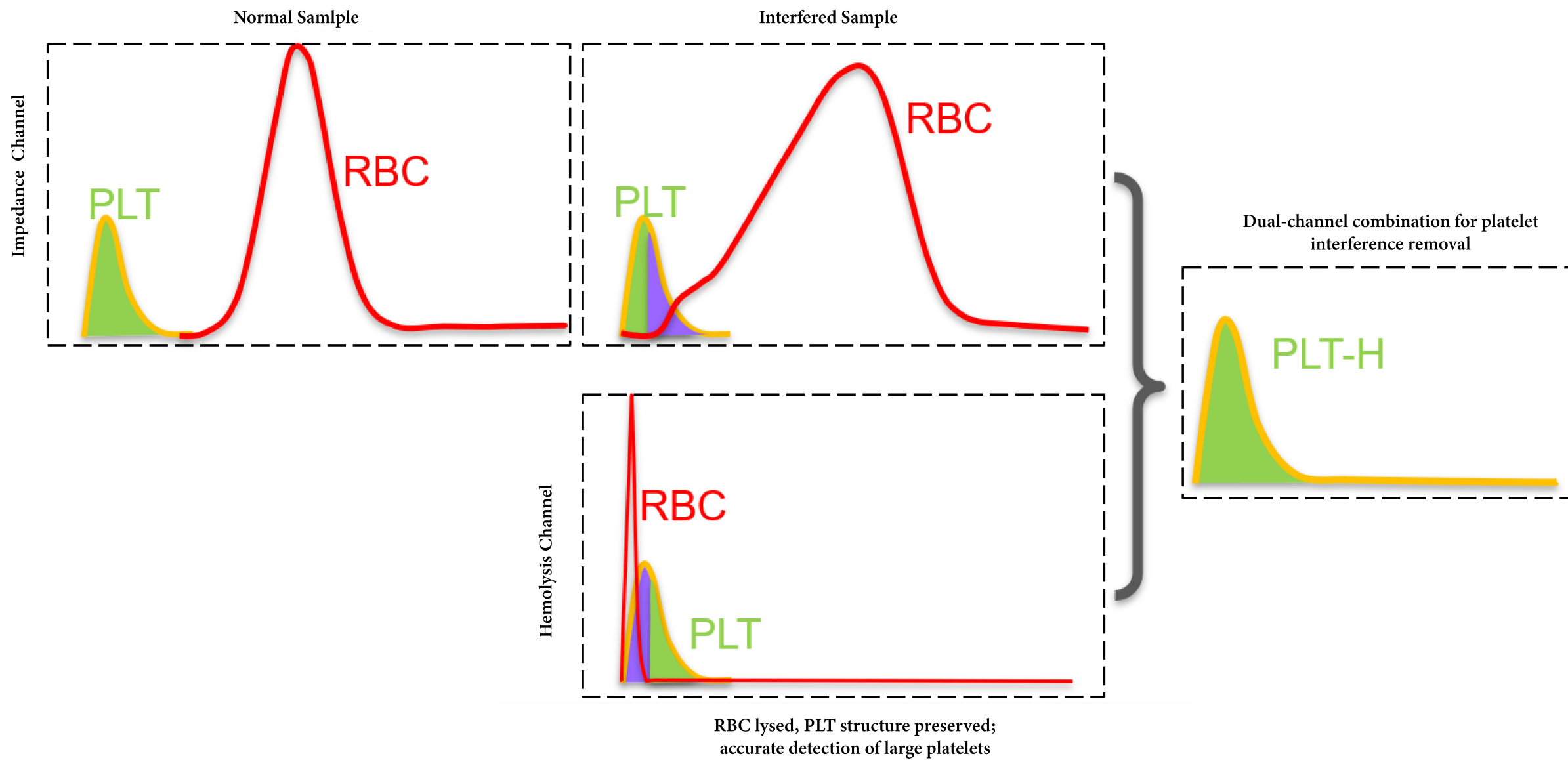

Figure 2 of the supplementary material

Supplement: Supplementary file 1 — Figure S1: jcla70185‐sup‐0001‐FiguresS1‐S2.pdf. Figure S2: jcla70185‐sup‐0001‐FiguresS1‐S2.pdf. [file JCLA-40-e70185-s002.pdf]
