# Supplementary material for: Integrated ESR and PLT‐H Measurement Using the BC‐6800 Plus Hematology Analyzer: A Comprehensive Analytical Evaluation
Source: J Clin Lab Anal. 2026 Feb 20;40(7):e70185. doi: 10.1002/jcla.70185 (PMC13052096; doi:10.1002/jcla.70185)
Supplement: Supplementary file 2 — Table S1: Background results of PLT‐H across multiple analyzer units and modes. Table S2: Repeatability of PLT‐H and ESR measurements across different modes and instruments. Table S3: Carryover evaluation of PLT‐H across within‐mode and between‐mode testing. Table S4: Accuracy of PLT‐H and PLT‐O compared to manufacturer‐assigned reference values. [file JCLA-40-e70185-s001.docx]

Supplementary Tables

Table S1. Background results of PLT-H across multiple analyzer units and modes

| Parameter | PLT-H | PLT-H | PLT-H | PLT-H |
| --- | --- | --- | --- | --- |
|  | 1# Maximum | 3# Maximum | 5# Maximum | 6# Maximum |
| AL WB CDR+ESR | 0 | 0 | 0 | 0 |
| AL WB CDR | 0 | 0 | 0 | 0 |
| OV WB CDR | 0 | 0 | 0 | 0 |
| PD CDR | 0 | 0 | 0 | 0 |
| Specification | ≤5 | ≤5 | ≤5 | ≤5 |
| Conclusion | PASS | PASS | PASS | PASS |

Background values of the PLT-H channel were tested using DS diluent blanks on four analyzer units (#1, #3, #5, and #6) across all operation modes (Auto Whole Blood CDR+ESR, Auto Whole Blood CDR, Open-vial Whole Blood CDR, and Predilute CDR). All results were 0 ×10⁹/L, meeting the predefined acceptance limit of ≤5 ×10⁹/L, confirming excellent background suppression

Table S2. Repeatability of PLT-H and ESR measurements across different modes and instruments

| Parameter | Mode | Instrument | MEAN | SD | CV | Requirement SD | Requirement CV | Result |
| --- | --- | --- | --- | --- | --- | --- | --- | --- |
| ESR | Auto whole blood CDR+ESR | 1# | 1.25 | 0.5 | 36.44% | 1.8 (ESR≤20 mm/h) | 9.00% (ESR>20 mm/h) | PASS |
|  |  |  | 13.99 | 0.1 | 1.01% | 1.8 (ESR≤20 mm/h) | 9.00% (ESR>20 mm/h) | PASS |
|  |  |  | 26.91 | 0.9 | 3.34% | 1.8 (ESR≤20 mm/h) | 9.00% (ESR>20 mm/h) | PASS |
|  |  | 4# | 38.33 | 0.1 | 0.32% | 1.8 (ESR≤20 mm/h) | 9.00% (ESR>20 mm/h) | PASS |
|  |  |  | 4.92 | 0.1 | 2.67% | 1.8 (ESR≤20 mm/h) | 9.00% (ESR>20 mm/h) | PASS |
|  |  |  | 13.82 | 1.5 | 11.16% | 1.8 (ESR≤20 mm/h) | 9.00% (ESR>20 mm/h) | PASS |
| PLT_H | Auto whole blood CDR | 1# | 971 | 12.9 | 1.33% | / | 4.00% | PASS |
|  |  |  | 120 | 3.0 | 2.50% | / | 4.00% | PASS |
|  |  |  | 94 | 2.0 | 2.16% | / | 4.00% | PASS |
|  |  |  | 228 | 3.6 | 1.56% | / | 4.00% | PASS |
|  |  |  | 35 | 1.1 | 3.04% | / | 4.00% | PASS |
|  |  | 4# | 204 | 4.0 | 1.95% | / | 4.00% | PASS |
|  |  |  | 93 | 2.1 | 2.29% | / | 4.00% | PASS |
|  |  |  | 761 | 14.6 | 1.91% | / | 4.00% | PASS |
|  |  |  | 285 | 5.6 | 1.98% | / | 4.00% | PASS |
|  |  |  | 38 | 0.9 | 2.52% | / | 4.00% | PASS |
|  | Open-vial whole CDR | 1# | 119 | 1.9 | 1.62% | / | 4.00% | PASS |
|  |  |  | 93 | 3.7 | 3.98% | / | 4.00% | PASS |
|  |  |  | 217 | 4.7 | 2.18% | / | 4.00% | PASS |
|  |  |  | 999 | 13.5 | 1.35% | / | 4.00% | PASS |
|  |  |  | 36 | 1.3 | 3.64% | / | 4.00% | PASS |
|  |  | 4# | 80 | 1.4 | 1.70% | / | 4.00% | PASS |
|  |  |  | 22 | 0.8 | 3.79% | / | 4.00% | PASS |
|  |  |  | 279 | 4.3 | 1.56% | / | 4.00% | PASS |
|  |  |  | 330 | 7.9 | 2.40% | / | 4.00% | PASS |
|  |  |  | 712 | 6.6 | 0.93% | / | 4.00% | PASS |
|  | Predilute CDR | 1# | 229 | 4.6 | 2.03% | / | 8.00% | PASS |
|  |  |  | 169 | 3.7 | 2.17% | / | 8.00% | PASS |
|  |  |  | 34 | 2.1 | 6.31% | / | 8.00% | PASS |
|  |  |  | 1321 | 14.4 | 1.09% | / | 8.00% | PASS |
|  |  |  | 89 | 2.7 | 3.07% | / | 8.00% | PASS |
|  |  | 4# | 149 | 4.4 | 2.99% | / | 8.00% | PASS |
|  |  |  | 32 | 1.7 | 5.27% | / | 8.00% | PASS |
|  |  |  | 84 | 2.7 | 3.21% | / | 8.00% | PASS |
|  |  |  | 281 | 5.8 | 2.08% | / | 8.00% | PASS |
|  |  |  | 1099 | 11.5 | 1.04% | / | 8.00% | PASS |

Repeatability was assessed using 10 consecutive replicates of multiple sample levels across Auto Whole Blood CDR+ESR, Auto Whole Blood CDR, Open-vial Whole Blood CDR, and Predilute CDR modes on analyzer units (#1 and #4). For ESR, results were evaluated at both ≤20 mm/h (SD ≤1.8 mm/h) and >20 mm/h (CV ≤9%). For PLT-H, CV thresholds were ≤4% (whole blood) and ≤8% (predilute). All results met acceptance limits, confirming stable repeatability for both ESR and PLT-H

Table S3. Carryover evaluation of PLT-H across within-mode and between-mode testing

| Combination | High value sample test mode | Low value sample test mode | PLT-H of 4# | PLT-H of 6# |
| --- | --- | --- | --- | --- |
| In mode: |  |  |  |  |
| 1 | Auto whole blood CDR | Auto whole blood CDR | 0.10% | 0.09% |
| 2 | Auto whole blood CDR +ESR | Auto whole blood CDR +ESR | 0.00% | 0.20% |
| 3 | Open-vial whole blood CDR | Open-vial whole blood CDR | 0.00% | 0.00% |
| 4 | Open-vial Predilute whole blood CDR | Open-vial Predilute whole blood CDR | 0.19% | 0.09% |
| Between modes: |  |  |  |  |
| 5 | Auto whole blood CDR +ESR | Open-vial whole blood CDR | 0.09% | 0.14% |
| 6 | Auto whole blood CDR +ESR | Open-vial Predilute whole blood CDR | -0.30% | -0.17% |
| 7 | Open-vial whole blood CDR | Auto whole blood CDR +ESR | 0.00% | -0.10% |
| 8 | Open-vial whole blood CDR | Open-vial Predilute whole blood CDR | 0.10% | 0.10% |
| 9 | Open-vial Predilute whole blood CDR | Auto whole blood CDR +ESR | -0.18% | 0.00% |
| 10 | Open-vial Predilute whole blood CDR | Open-vial whole blood CDR | 0.00% | -0.10% |
| Specification | | | ≤1.0% | ≤1.0% |
| Conclusion | | | PASS | PASS |

Carryover was calculated by sequentially testing high-value platelet samples (>900×10⁹/L) and low-value samples (<30×10⁹/L) across two analyzer units (#4 and #6) under both within-mode and between-mode conditions. All carryover values ranged from −0.30% to 0.20%, well below the acceptance threshold of ≤1.0%, indicating negligible residual signal and minimal risk of cross-sample contamination

Table S4. Accuracy of PLT-H and PLT-O compared to manufacturer-assigned reference values

| Sample | | PLT_O | PLT_H |
| --- | --- | --- | --- |
| Sample 1 | Reference Value | 233.9 | 233.9 |
| Sample 2 | Reference Value | 176.6 | 176.6 |
| Sample 3 | Reference Value | 178.6 | 178.6 |
| Sample 1 | Test 1 | 225 | 231 |
|  | Test 2 | 227 | 222 |
| Sample 2 | Test 1 | 167 | 181 |
|  | Test 2 | 166 | 178 |
| Sample 3 | Test 1 | 196 | 193 |
|  | Test 2 | 189 | 196 |
| Sample 1 | Test 1 (d%) | -3.81% | -1.24% |
|  | Test 2 (d%) | -2.95% | -5.09% |
| Sample 2 | Test 1 (d%) | -5.44% | 2.49% |
|  | Test 2 (d%) | -6.00% | 0.79% |
| Sample 3 | Test 1 (d%) | 9.74% | 8.06% |
|  | Test 2 (d%) | 5.82% | 9.74% |
| Acceptance Criteria | | ±20.0% | ±20.0% |
| Conclusion | | Pass | Pass |

Relative bias (d%) was calculated as: (Test value − Reference value) / Reference value × 100%. All results met the predefined acceptance criterion of ±20%.

Three reference samples were tested in duplicate on both PLT-H and PLT-O channels. Relative bias (%) was calculated against target values, with an acceptance criterion of ±20%. Both PLT-H and PLT-O results met this requirement, but PLT-H showed smaller biases and tighter distributions. This demonstrates higher accuracy and consistency of the hybrid PLT-H method compared with PLT-O
